# Developing a tool for measuring postpartum women's experiences of respectful maternity care at a tertiary hospital in Kumasi, Ghana

**DOI:** 10.1016/j.heliyon.2020.e04374

**Published:** 2020-07-10

**Authors:** Veronica Millicent Dzomeku, Adwoa Bemah Boamah Mensah, Kweku Emmanuel Nakua, Pascal Agbadi, Jody R. Lori, Peter Donkor

**Affiliations:** a Department of Nursing, Faculty of Allied Health Sciences, College of Health Sciences, Kwame Nkrumah University of Science and Technology, Kumasi, Ghana; b Department of Epidemiology and Biostatistics, School of Public Health, Kwame Nkrumah University of Science and Technology, Kumasi, Ghana; c School of Nursing, University of Michigan, USA; d Department of Surgery, School of Medical Sciences, Kwame Nkrumah University of Science and Technology, Kumasi, Ghana; e Komfo Anokye Teaching Hospital, Kumasi, Ghana

**Keywords:** Public health, Women's health, Pediatrics, Sociology, Respectful maternity care, Scale development, Exploratory factor analysis, 23i-RMC, Ghana

## Abstract

The authors of this paper are involved in a 5 years respectful maternity care (RMC) project at a tertiary healthcare facility in Kumasi, Ghana that seeks to change the culture of disrespect and abuse in maternity care practice, with a sub-objective of determining its impact on how midwives provide quality maternity care services in this healthcare facility. To achieve this objective, respectful maternity care must be conceptualized and measured. Our literature search revealed that a Ghanaian version tool that measures women's experiences of respectful maternity care is non-existent. Thus, this study aims to construct a scale that measures childbearing women's experiences of respectful maternity care during childbirth and the immediate postpartum period in the study setting. We surveyed 263 postpartum women with a draft scale we have developed. This scale had 42 questions that sought to measure postpartum women's experiences of respectful maternity care in a tertiary health facility in Kumasi. The scale development went through processes of exploratory factor analyses (EFA) and inter-item reliability tests. The EFA was done using SPSS-21. Through series of EFA, we have created a 23 items RMC scale (23i-RMC) with three main factors labelled as follows: Verbal abuse-free, Discriminatory-free and Dignified care (VADDC), Physical and Psychological Abuse-free care (PPAC), and Compassionate Care (CC). The Cronbach's Alpha of the 23i-RMC is 0.945 and those of the individual domains greater than the 0.70 minimum threshold, suggesting that there is greater reliability among the items in the scale and the subscales. This 23i-RMC scale is useful for assessing postpartum women's experiences of RMC in the study setting. We recommend the use and validation of the newly developed respectful maternity care scale in other healthcare facilities in Ghana.

## 1 Introduction

Along with other effective and efficient policies and programs, many low-and-middle-income countries (LMICs) are reducing their unacceptably high maternal and neonatal death rates through the promotion and implementation of healthcare facility-based deliveries with skilled birth attendants [1, 2, 3, 4, 5]. Despite the positive contribution of the facility-based delivery intervention, a growing body of evidence indicates that many childbearing women are becoming victims of disrespectful and abusive care in the hands of maternity care providers in countless healthcare facilities in LMICs [6, 7, 8]. Freedman defined disrespectful and abusive care as the “interactions or facility conditions that local consensus deem to be humiliating or undignified, and those interactions or conditions that are experienced as or intended to be humiliating or undignified” [9].

Given the evidence that disrespect and abusive care (D&AC) practices destroy the trust of childbearing women in healthcare systems that can result in adverse maternal and neonatal outcomes [8], programs that seek to promote respectful maternity care (RMC) services during pregnancy, childbirth, and the immediate postpartum period have received massive scholarly and donor support [10, 11, 12, 13, 14, 15, 16]. RMC is the recognition and administering of maternity care services that promote the rights of the childbearing woman to quality healthcare devoid of any form of abuse (physical, psychological, and verbal), discrimination, dehumanization, humiliation, and disrespect [17].

The first steps to eradicate D&AC through the promotion of RMC involves the conceptualization and measuring of women's experiences of the phenomenon. This has been well documented in some studies, in which scholars have designed measurement tools that captures either the full spectrum of women's experiences of RMC or aspects of it [18, 19, 20, 21]. The mothers on respect (MOR) index, for instance, is an RMC measurement tool that assesses how the client-provider interactions affect childbearing women's sense of comfort, behaviour and perceptions of racism or discrimination [21]. The same authors have created another scale, “Mother's Autonomy in Decision Making” (MADM), that measures women's satisfaction with their decision making during maternity care [20]. The MOR and the MADM were developed within the context of Canada and USA. Other RMC scales were developed within the context of LMICs [18, 19], with one originating from Ethiopia [19]. Although all these scales are tools for measuring women's experiences of RMC, the subscales and items of the different scales have notable variations in the way certain aspect of the phenomenon were conceptualized. These observed variations were mainly context-specific, making it an important criterion in the design and measuring of RMC within a Ghanaian context.

The authors of this paper are involved in a 5 years RMC project at a tertiary healthcare facility in Kumasi, Ghana that seeks to change the culture of disrespect and abuse in maternity care practice, with a sub-objective of determining its impact on how midwives provide quality maternity care services in this healthcare facility. To achieve this objective, RMC must be conceptualized and measured. Our literature search revealed that a Ghanaian version tool that measures women's experiences of RMC is non-existent. Thus, this study aims to construct a scale that measures childbearing women's experiences of RMC during childbirth and the immediate postpartum period in the study setting.

## 2 Materials and methods

### 2.1 Study setting

Our study is situated in a tertiary health facility in Kumasi, located in the Ashanti region of Ghana. This facility provides healthcare services to patients across the country and has a bed capacity of about 1200 and a staff strength of about 3000. It is the main referral hospital for the Ashanti, Brong Ahafo, Bono East, Ahafo, Western, Western North, the five northern regions (Northern, Upper East, Upper West, North East, Savannah), and neighbouring countries. It has twelve (12) directorates (departments) one of which is the Obstetrics and Gynaecology (O &G) directorate, which has four labour wards. In 2018, the hospital recorded an estimated 4792 Spontaneous vaginal deliveries, an estimated 123 maternal deaths, and 61 neonatal deaths [KATH O & G Records, 2019].

### 2.2 Expert review and instrument

We obtained a 60-items draft RMC scale from an RMC research team in Ethiopia [19]. The first four authors reviewed each of the items and selected 42 items that were contextually applicable to Ghana. The authors have extensively undertaken qualitative research among trainee midwives and practising midwives' descriptions of how they provide respectful maternity care and postpartum women's experiences of respectful maternity care in the study setting [6, 22, 23], so their expertise were relevant in selecting the items that best capture the experiences of women receiving intrapartum care services in the health facility. Also, the third and fourth authors provided technical expertise in the choice of extraction and rotation methods to use in developing the tool because of their research and academic expertise in biostatistics, public health, and health promotion.

### 2.3 Sampling and data collection

We used a convenient sampling technique to recruit the study participants from the postpartum unit at the hospital. Trained research assistants (RAs) visited the postpartum unit twice a week between April and August 2019 and approached the women and explain the purpose of the research to obtain written consent before administering the questionnaires. The RAs were bachelor's degree holders, fluent in English, and native “Twi” speakers. The questionnaire comprises of sections on demographic details of the participants and the 42-item RMC draft scale. The RAs successfully administered 270 questionnaires, seven of which were partially filled and were excluded. Respondents were asked to rank their RMC experiences on a 5-point Likert scale: strongly agree (5), agree (4), don't know (3), disagree (2), strongly disagree (1).

### 2.4 Data management and analysis

The collected data were entered and managed in SPSS version 21. Given that we selected the respondents based on convenient sampling technique, we used the principal axis factoring (PAF), which is recommended when the data violates assumptions of multivariate normality [24]. We chose varimax with Kaiser normalization as the rotation method for its superiority among other orthogonal factor rotation methods in producing a simplified factor structure [25]. We set the communality and factor loading thresholds at 0.50 to help retain items that best measure the RMC following the guidelines of Hair et al [25]. Results from the KMO and Bartlett's Test of sphericity determined the sample adequacy and the suitability of the data for factor analysis. We performed an inter-item reliability test to determine the internal consistency among the items and the domains of the newly developed RMC scale. We assess the validity of the tool by performing correlation analysis among the subscales, and we used the Kruskal Wallis test to determine the relationship between the RMC and its components and respondents' level of education.

## 3 Ethical consideration

We sought and obtained ethical clearance from the Committee on Human Research, Publication, and Ethics (CHRPE) at the Kwame Nkrumah University of Science and Technology (KNUST) (reference number: CHRPE/AP/181/18) and the Komfo Anokye Teaching Hospital Institutional Review Board (reference number: RD/CR17/289). Respondents were interviewed in the comfort of their homes after they have given their verbal consent for visitation and written consent for questionnaire administration. We anonymised information that may reveal details of the participants to third parties. Participants were informed of their right to voluntary participation.

## 4 Results

### 4.1 Summary statistics of respondents' profile

Majority of the respondents were within the age group of 25–29 years (32.7%), had no formal education (36.4%), were Christians (82.2%), and were uniparous (35.9%) (see Table 1).

**Table 1:** Profile of respondents.

Variable	N (%)
**Age**	
15–19	18 (6.9)
20–24	35 (13.5)
25–29	85 (32.7)
30–34	80 (30.8)
35–39	35 (13.5)
40+	7 (2.7)
Missing	2

**Education**	
No education	95 (36.4)
Primary	64 (24.5)
JHS	11 (4.2)
SHS	51 (19.5)
Tertiary	40 (15.3)
Missing	2

**Religion**	
Christian	212 (82.2)
Islam	46 (17.8)
Missing	5

**Parity**	
1	92 (35.9)
2	56 (21.9)
3	52 (20.3)
4+	56 (21.9)
Missing	7

JHS: Junior Secondary School; SHS: Senior Secondary School.

### 4.2 Summary statistics on sampling adequacy and the number of extracted factors

We reported summary statistics of sampling adequacy and the number of extracted factors at all stages of the EFA (see Table 2). The newly developed scale consists of three domains and 23 items (see Table 3). Nineteen (19) items were dropped from the draft scale for the following reasons: low communality scores and cross loading on two factors (see Table 4). The Kaiser-Meyer-Olkin value of the final scale was 0.932, which is above the minimum threshold of 0.6 (Kaiser 1970, 1974) and Bartlett's Test of Sphericity (Bartlett 1954) reached statistical significance, supporting the factorability of the correlation matrix (see Table 2). The three-factor solution explained a total of 78.39% of the variance, with factor one, two, and three contributing 38.49%, 30.82%, and 8.08% of the explained variance, respectively (see Table 2). We labelled the extracted three factors as Verbal abuse-free, Discriminatory-free, and Dignified care (VADDC), Physical and Psychological Abuse-free care (PPAC), and Compassionate Care (CC). There was a weak negative correlation between VADDC and PPAC (ρ = -0.153, p ≤ 0.05), moderate positive correlation between VADDC and CC (ρ = 0.639, p ≤ 0.01), and weak positive correlation between PPAC and CC (ρ = 0.164, p ≤ 0.01), indicative of construct validity of the 23i-RMC scale.

**Table 2:** Summary statistics on sampling adequacy and the number of extracted factors.

	Initial	Final
Items in the scale	42	23
Items deleted	0	19
Factors Extracted	6	3
Sample size	263	263
Total variance explained	64.41%	78.28
KMO	91.5%	93.2%
Bartlett's test of sphericity	χ^2^ = 11032.73∗	χ^2^ = 7904.56
Degree of freedom	861	253

∗p < 0.001.

**Table 3:** Rotated factor matrix for the 23-item RMC scale.

Statements	Factors			Com.
	1	2	3	
**VADDC** 1: I was detained in the facility because I don't have enough money to pay (R)	0.789			0.669
**VADDC** 2: I was left alone after delivery (R)	0.820			0.706
**VADDC** 3: Service provision was delayed due to supplies even if it is available in the facility (R)	0.906			0.858
**VADDC** 4: 9 I felt there was inappropriate touching of genital/thighs during an exam (R)	0.872			0.786
**VADDC** 5: The health worker didn't mention anything that she/he is performing (R)	0.853			0.792
**VADDC** 6: The health workers shouted on me because I touched her hands during contraction (R)	0.930			0.901
**VADDC** 7: Some health workers insulted me and my companions (R)	0.918			0.881
**VADDC** 8: I felt like the health workers tried to move things along for their own convenience (R)	0.832			0.788
**VADDC** 9: Some of the health workers do not treat me well because of some personal attribute (economic, education, residence, language etc) (R)	0.895			0.840
**VADDC** 10: My treatment was delayed because I couldn't pay for the services (R)	0.898			0.855
**VADDC** 11: My companions were allowed to enter the delivery room during delivery	0.754			0.620
**PPAC 1:** Some health providers showed me an intimidating gesture (R)		0.873		0.779
**PPAC 2:** The health provider slapped me during delivery(R)		0.904		0.836
**PPAC 3:** The health provider applied excessive force to pull the baby out (R)		0.893		0.806
**PPAC 4:** The health worker Stitched me (applied episiotomy) without anaesthesia (R)		0.751		0.575
**PPAC 5:** I felt that I was physically abused during delivery(R)		0.858		0.769
**PPAC 6:** I felt that I was sexually abused during delivery (R)		0.940		0.903
**PPAC 7:** I was tied to delivery coach during delivery (R)		0.890		0.815
**PPAC 8:** I was uncovered unnecessarily (R)		0.925		0.864
**PPAC 9:** I was kept waiting for a long time before receiving service (R)		0.818		0.673
**CC 1:** The health workers show active involvement during contraction			0.772	0.785
**CC 1:** The health workers provided coaching on breathing and relaxation			0.734	0.770
**CC 1:** The health workers were talking positively about pain and relief			0.578	0.529
**Eigenvalues**	**10.90**	**6.33**	**1.27**	

Com.: Communality.

**Table 4:** Items deleted for cross-loading and low communality.

Items deleted	Reason for deletion
The health worker responded to my needs whether or not I asked	Low communality
The health worker encouraged me to open my legs during labour	Low communality
I felt that health workers cared for me with a kind approach	Low communality
I was told that I can refuse a procedure if I don't like it	Low communality
My consent was requested for all procedures performed	Low communality
The health workers talked to me and my companions politely	Low communality
The health workers speak to me in a language that I can understand	Low communality
Health workers treat all patients equally	Low communality
During delivery, the health worker draped or covered me to protect my privacy	Low communality
The health provider greeted me and my companions	Low communality
The health provider called me by my name	Low communality
The health provider talked to me in a friendly manner	Low communality
All health workers treated me with respect as an individual	Low communality
The health workers used reassuring touch	Low communality
The couches were separated by privacy screens during an examination	Low communality
The counselling sessions were held in a private area	Low communality
The health worker showed her concern and empathy	Low communality
The health workers show active involvement during contraction	Low communality
The health workers provided coaching on breathing and relaxation	Low communality
The health workers were talking positively about pain and relief	Low communality
My privacy is protected during labour and delivery	Cross loaded on two factors
The health provider helped me to try different delivery positions	Cross loaded on two factors

### 4.3 Summary and reliability statistics of the 20i-RMC scale

We performed basic descriptive and inter-item reliability analyses for the sub-scale and the full 23i-RMC scale (see Table 5). The Cronbach's Alpha of the 23i-RMC scale is 0.945 and that of the individual domains are: VADDC (11 items), α = 0.974; PPAC (9 items), α = 0.968; CC (3 items), α = 0.865. These Cronbach's Alpha values are above the minimum threshold of 0.70, suggesting that there is greater reliability among the items on the main 23i-RMC scale and in the two sub-scales.

**Table 5:** Reliability statistics for the RMC Scale.

	M [SD]	Min-Max	Cronbach's Alpha	Alpha if an item is deleted
**VADDC**	**42.10 [15.0]**	**11.0–55.0**	**0.974**	
VADDC 1	3.98 [1.49]			0.974
VADDC 2	3.94 [1.45]			0.973
VADDC 3	3.82 [1.51]			0.971
VADDC 4	3.88 [1.50]			0.972
VADDC 5	3.89 [1.50]			0.972
VADDC 6	3.81 [1.58]			0.970
VADDC 7	3.84 [1.56]			0.970
VADDC 8	3.86 [1.46]			0.972
VADDC 9	3.87 [1.55]			0.971
VADDC 10	3.86 [1.55]			0.971
VADDC 11	3.36 [1.64]			0.976
**PPAC**	**21.94 [13.48]**	**9.0–45.0**	**0.968**	
PPAC 1	2.29 [1.63]			0.964
PPAC 2	2.36 [1.73]			0.962
PPAC 3	2.30 [1.66]			0.963
PPAC 4	2.39 [1.69]			0.969
PPAC 5	2.56 [1.71]			0.965
PPAC 6	2.47 [1.68]			0.961
PPAC 7	2.42 [1.69]			0.963
PPAC 8	2.45 [1.63]			0.962
PPAC 9	2.70 [1.69]			0.967
**CC**	**12.35 [2.75]**	**3.0–15.0**	**0.865**	
CC 1	4.11 [1.00]			0.775
CC 2	4.12 [1.05]			0.771
CC 3	4.12 [1.05]			0.880
**23i-RMC**	**76.39 [23.76]**	**20.0–100.0**	**0.945**	

M: mean; SD: Standard Deviation; Min.-Max.: Minimum and Maximum scores.

### 4.4 The relation between respondents' education and RMC

The mean and SD values of the main scale and the sub-scales suggest that the data is skewed. Thus, we performed a normality test to ascertain whether to perform a parametric or a non-parametric test to examine if RMC—as measured by the newly developed scale—has any relationship with respondents' level of education. We used the Kolmogorov-Smirnov statistic to test for normality of the distribution of scores. The Kolmogorov-Smirnov statistic was significant (p = 0.00), indicating violation of the assumption of normality. Therefore, we performed a Kruskal Wallis test, a non-parametric version of One-Way ANOVA, to assess the relationship between the level of education and RMC.

Before the test of association, we transformed the scores of each respondent on the main and subscales into a percentage scale for easy interpretation. To create the percentage score for respondents on each subscale and the main 23i-RMC, we subtracted the minimum score from the score of the respondent divided by the difference between the actual maximum score and the actual minimum score multiplied by 100 [((Respondent's score-minimum score)/(Actual maximum score-minimum score)∗100)].

A Kruskal-Wallis test revealed a statistically significant difference in respondent's experiences of RMC across five different education level (no education, n = 95; primary, n = 11; JHS, n = 64; SHS, n = 51; Tertiary, n = 40), χ^2^ (4, n = 261) = 109.14, p = 0.00. The postpartum women who had no formal education recorded the highest median score (Md = 79.35%) compared to the other women with different levels of education: primary, Md = 56.52%; JHS, Md = 56.52; SHS, Md = 57.61%; Tertiary, Md = 60.33%. The result suggests that postpartum women with no formal education rated their experiences of RMC at the hospital higher than those with some form of education.

We further assessed the relationship between each of the subscales of 23i-RMC and respondents' level of education. First, we observed a statistically significant difference in respondent's experiences of VADDC across five different education level, χ^2^ (4, n = 261) = 15.82, p = 0.003. The women who had a tertiary level education recorded the highest median score (Md = 97.73%) compared to the other women with different levels of education: no education, Md = 81.82%; primary, Md = 90.91; JHS, Md = 71.59%; SHS, Md = 75.00%. The results suggest that more women with tertiary level education rated that they had experienced a non-discriminatory, privacy-sensitive, and consent seeking care.

Secondly, we observed a statistically significant difference in respondent's experiences of PPAC across five different education level, χ^2^ (4, n = 261) = 116.13, p = 0.000. The postpartum women who had no formal education recorded the highest median score (Md = 77.78%) compared to the other women with different levels of education: primary, Md = 0.00%; JHS, Md = 6.94; SHS, Md = 8.33%; Tertiary, Md = 0.00%. The result suggests that postpartum women with no formal education rated their experiences of PPAC at the hospital higher than those with some form of education. The results suggest that more women with some form of education unsatisfactorily rated PPAC maternity care. However, women's PPAC scores across the level of education are generally low, suggesting that many of the respondents have reported the type of intrapartum care received at the hospital to be physically and psychologically abusive.

Lastly, we observed a statistically significant difference in respondent's experiences of CC across five different education levels, χ^2^ (4, n = 261) = 24.47, p = 0.000. The postpartum women who had no formal education recorded a higher median score (Md = 91.67%) than other women with at least a primary school level education, all with a median score of 75.00. The result suggests that postpartum women with no formal education rated their experiences of CC at the hospital higher than those with some form of education.

## 5 Discussion

The study aimed to develop a tool to measure RMC in the study setting. Through EFA with a draft questionnaire with 42 items, we have now developed a scale to measure RMC.

The newly developed RMC scale, 23i-RMC, has three subscales—VADDC, PPAC, and CC. This suggests that postpartum women who visited the hospital perceived RMC as compassionate care and one devoid of abuse, discrimination, and disrespect. We included questions on privacy in the draft scale, but we had to drop them because of their low communality values and issues of cross-factor loadings. This could mean that the postpartum women did not perceive privacy as a critical component of RMC, but this may have to be further investigated. The summary statistics revealed that the respondents recorded a low score on the PPAC, suggesting that many of them might have been physically and psychologically abused during childbirth.

The items and components in the 23i-RMC share some similarities with the one designed by the Ethiopian team and other recently developed scales [18, 19, 20, 21]; however, there are some noteworthy variations. For instance, though questions on detainment in a hospital due to non-payment of service fees, improper vagina examination, and consent seeking were included in the 23i-RMC scale due to their high factor loadings and communality values in our scale, the same questions were dropped from the scale developed by Sheferaw et al due to their low communality values and low factor loadings [19]. The few other developed scales also did not have questions on detainment due to non-payment of service fees [18, 20, 21]. The validity and reliability of these questions only confirm what some studies from Ghana have revealed about the regular detainment of some postpartum women after delivery due to non-payment of service fees in healthcare facilities across the country [11, 23, 26]. These variations between the 23i-RMC and existing RMC scales prove that context implications of RMC should be considered when seeking to measure it.

We assessed whether there were differences in respondents' perceive RMC experiences across the level of education. Generally, respondents with no formal education positively rated higher their experiences of RMC compared to those with some form of formal education. There is a possibility that postpartum women with no formal education did not have high expectations compared to their counterparts with some form of formal education. Our result is consistent with studies conducted in Kenya [27] and the Netherlands [28]. These studies found that women with a higher level of education rated their maternity care experiences to be unsatisfactory.

## 6 Conclusion

The study objective of developing an RMC scale to measure postpartum women's perception of RMC at the KATH was achieved. Through series of EFA, we have created a 23 items RMC scale (23i-RMC) with three main factors labelled as follows: Verbal abuse-free, Discriminatory-free and Dignified care (VADDC), Physical and Psychological Abuse-free care (PPAC), and Compassionate Care (CC). Postpartum women experiences of RMC at the facility differed based on their educational qualification, with the women with no formal education rating their experiences of RMC on the full scale and two of the subscales (PPAC and CC) at the hospital higher than those with some form of education. However, more women with tertiary level education rated their experiences on the VADDC subscale higher than those with lower or no formal education. Generally, women's PPAC scores across the level of education are low, suggesting that many of the respondents have reported the type of intrapartum care received at the hospital to be physically and psychologically abusive.

This is the first study to the best of our knowledge from Ghana on RMC tool development, and the 23i-RMC scale proved valid and reliable in measuring RMC among women who delivered at the study setting. Nonetheless, we recommend that further exploratory and confirmatory factor analyses should be performed with a larger sample of postpartum women in the study setting and from other healthcare facilities in the region and across the country.

## Declarations

### Author contribution statement

V. M. Dzomeku and Adwoa B. B. Mensah: Conceived and designed the experiments; Performed the experiments; Contributed reagents, materials, analysis tools or data; Wrote the paper.

E. K. Nakua: Conceived and designed the experiments; Performed the experiments; Analyzed and interpreted the data; Contributed reagents, materials, analysis tools or data; Wrote the paper.

J. R. Lori and P. Donkor: Conceived and designed the experiments; Performed the experiments; Wrote the paper.

P. Agbadi: Analyzed and interpreted the data; Wrote the paper.

### Funding statement

V. M. Dzomeku was supported by 10.13039/100000061Fogarty International Center (K43TW011022).

### Competing interest statement

The authors declare no conflict of interest.

### Additional information

No additional information is available for this paper.
